# mHealth To Promote Monitoring and Self‐Regulation Among Caregivers of People With Dementia: A Systematic Review

**DOI:** 10.1002/pchj.70092

**Published:** 2026-04-05

**Authors:** Felipe Soto‐Pérez, Camila Ruy Castilla, Madalin M. Deliu

**Affiliations:** ^1^ Depto. Personalidad, Evaluación y Tratamiento Psicológicos Universidad de Salamanca Salamanca Spain; ^2^ INICO: Instituto Universitario de Integración en la Comunidad Salamanca Spain; ^3^ IBSAL: Instituto de Investigación Biomédica de Salamanca Salamanca Spain; ^4^ Universidad Católica de Ávila Ávila Spain

**Keywords:** caregivers' burden and wellbeing, dementia caregiving, mHealth, monitoring features, self‐regulation

## Abstract

Dementia caregiving imposes substantial emotional, physical, and financial burdens, underscoring the urgent need for scalable and accessible support interventions. This systematic review evaluates the role of mobile health (mHealth) applications in enhancing caregivers' self‐regulation, with a particular focus on the monitoring component, and their impact on caregivers' burden and wellbeing. Twenty‐four studies published between 2019 and 2025 were reviewed, analyzing intervention models, the presence and implementation of monitoring strategies, and overall app efficacy. The findings reveal a variety of mHealth approaches, including psychoeducational tools, social and peer support features, and mindfulness‐based therapies. Monitoring mechanisms—such as behavior tracking, emotional self‐assessments, reflective prompts, and chatbot‐based feedback—emerged as pivotal elements for activating the self‐regulation process. These tools supported caregivers in recognizing stressors, evaluating caregiving strategies, and making adaptive changes, thereby enhancing emotional resilience and caregiving efficacy. However, persistent challenges such as declining user engagement and variability in digital literacy highlight the need for more adaptive, user‐centered designs. This review emphasizes the transformative potential of mHealth monitoring in dementia caregiving and calls for future research to standardize evaluation metrics, personalize interventions, and promote long‐term engagement and accessibility for diverse caregiver populations.

## 1 Introduction

Dementia, classified as *Major Neurocognitive Disorder* in the *DSM‐5‐TR*, is a progressive condition that impairs key cognitive functions, including memory, attention, visuospatial abilities, language, and executive functioning (American Psychiatric Association 2022). Currently, more than 55 million people worldwide live with dementia, a number projected to rise to 78 million by 2030 and 139 million by 2050, driven by aging populations and increased life expectancy (World Health Organization [WHO] 2021). As a major contributor to global disability‐adjusted life years (DALYs), dementia poses a critical public health challenge among non‐communicable diseases (Nichols et al. 2022). It significantly limits individuals' ability to perform daily activities and maintain independence, impacting not only individuals but also their caregivers. Family members and informal caregivers—who often provide the majority of dementia‐related care—experience significant physical, emotional, and financial strain, highlighting the far‐reaching impact of dementia on households and healthcare systems alike (Riffin et al. 2019).

In most countries, family carers—spouses, children, and friends—are responsible for 50%–90% of dementia caregiving, often without formal training or support (Alzheimer's Association 2019; Pérez Díaz et al. 2020; Wang et al. 2022). This role often leads to chronic stress, declining health, and reduced quality of life, largely due to the complex and demanding nature of caregiving (Rathnayake et al. 2020; Wimo et al. 2023). Research suggests that caregivers frequently neglect their own health, prioritizing the needs of the care recipient over their own wellbeing (NICE 2018; Schulz and Sherwood 2008). This neglect can impair caregivers' ability to monitor their health and regulate their behaviors, emotions, and coping strategies, further exacerbating stress and burnout (Rathnayake et al. 2019; Wiegelmann et al. 2021).

Caregiving entails multifaceted challenges that require targeted psychosocial interventions (Wiegelmann et al. 2021). Its demands can reduce caregivers' autonomy (Panzeri et al. 2024; Rathnayake et al. 2019), highlighting the importance of strategies that foster self‐awareness and health monitoring. Self‐regulation—monitoring, evaluating, and adapting one's behaviors and emotions—may be key for caregivers' wellbeing (Behrouian et al. 2021; Panzeri et al. 2024). mHealth tools that enable self‐regulation through feedback, goal‐setting, and adaptive guidance (Rathnayake et al. 2019) offer accessible and cost‐effective support (Laver et al. 2020), enhancing coping, resilience (Panzeri et al. 2024), and health autonomy (Chi and Demiris 2015).

However, evidence on how mHealth interventions influence caregivers' health and caregiving practices remains limited. Prior reviews focus mainly on usability and general outcomes (Jagoda et al. 2023; Ye et al. 2023; Zou et al. 2024) or health‐information seeking (Rathnayake et al. 2019), noting barriers to development and a lack of rigorous evaluation; none have examined effects on burden and wellbeing. Given the rising prevalence of dementia (WHO 2021) and rapid technological growth (Jagoda et al. 2023; Zou et al. 2024), updated and methodologically robust reviews are needed.

This review addresses these gaps by examining recent (2019–2025) mHealth interventions for dementia caregivers, focusing on technology‐based self‐regulation—particularly monitoring—and its impact on stress management, caregiving practices, and wellbeing to inform more effective and sustainable interventions. To frame this study, we first outline the challenges faced by caregivers and the relevance of self‐regulated learning—particularly monitoring. We then review the current landscape of mHealth applications to contextualize mHealth‐based self‐management, identify gaps in existing research, and underscore the need for the present study.

### 1.1 Challenges Faced by Family Caregivers of People With Dementia

Caregivers' burden encompasses physical, emotional, social, and financial strain, contributing to frustration, low self‐esteem, and feelings of helplessness (Phillips et al. 2023; Riffin et al. 2019; Quinn et al. 2019). This burden can also influence decisions to institutionalize care recipients (Riffin et al. 2019), and is linked to negative health outcomes such as depression, anxiety, cardiovascular disease, loneliness, and social isolation, all of which reduce quality of life (El‐Hayek et al. 2019; Hébert et al. 2000; Tkatch et al. 2017; Zarit et al. 1986). As a result, caregivers often neglect their own health, which impairs their ability to manage stress, regulate emotions, and maintain overall wellbeing (NICE 2018; Schulz and Sherwood 2008).

Despite these challenges, caregiving can also be fulfilling, fostering personal growth, deeper relationships, and a sense of purpose (Brown and Brown 2014; Yu et al. 2018). Resilience factors, such as self‐efficacy and personal mastery, would help buffer stress and improve mental health (Durán‐Gómez et al. 2020; Harmell et al. 2011). Thus, effective interventions should address both burdens and rewards by promoting self‐regulation strategies, enhancing resilience, and supporting caregivers' long‐term wellbeing.

### 1.2 The Role of Self‐Regulation and Monitoring in Dementia Caregiving

Self‐monitoring—the capacity to monitor, evaluate, and adapt behaviors and emotions to achieve specific goals (Carver and Scheier 1981; Inzlicht et al. 2021)—is essential for managing caregiving demands, as it reduces stress, enhances emotional wellbeing, and fosters resilience (Behrouian et al. 2021; Panzeri et al. 2024). Specifically, interventions incorporating intensive monitoring mechanisms significantly optimize health outcomes and goal attainment among caregivers (Carver and Scheier 1981).

Monitoring enhances the observation of behavioral, emotional, and physiological states, enabling caregivers to identify discrepancies between their current status and wellbeing goals. This process triggers corrective actions and more compassionate care (Schulman‐Green et al. 2021), while facilitating stress management, time organization, and social support seeking (Baumeister and Heatherton 1996). Consequently, the resulting adaptive capacity allows for flexible responses to evolving environmental demands, thereby preserving long‐term wellbeing (Pinquart and Sörensen 2003; Wiegelmann et al. 2021).

Self‐regulation serves as a fundamental preventive pillar for physical and mental health. Mastery of these skills facilitates emotional management, mitigates burnout risk, and strengthens the bond with care recipients (Panzeri et al. 2024). Specifically, monitoring and adjusting health behaviors prevents chronic stress from progressing into severe pathologies such as depression, anxiety, or cardiovascular disease (Reed et al. 2020; Sardella et al. 2022). This awareness enables caregivers to implement preventive measures in critical domains such as sleep, nutrition, and physical activity, ensuring that personal wellbeing remains aligned with caregiving values and goals (Heatherton 2011; Schulz and Sherwood 2008).

In summary, self‐monitoring enhances caregivers' awareness and resilience by facilitating proactive adjustments (Schulman‐Green et al. 2021); however, caregiving demands often hinder sustained practice, increasing vulnerability to burnout (Behrouian et al. 2021; Panzeri et al. 2024). To address this challenge, mHealth interventions have the potential to support continuous self‐regulation by optimizing wellbeing monitoring.

### 1.3 Advancements in mHealth Technology and Its Relevance for Caregivers' Monitoring

Mobile health (mHealth) technologies, that is, as the use of mobile devices such as smartphones and tablets to deliver healthcare and preventive services (American Psychological Association 2025), offer opportunity to mitigate caregiving challenges. These ecosystems integrate applications, wearable devices, and videoconferencing platforms (Angelopoulou et al. 2022), providing scalable, cost‐effective, and accessible tools to meet the demands of family caregivers across diverse settings (Goodridge et al. 2021; Kuo et al. 2022). Although digital literacy and connectivity barriers remain critical obstacles for implementation (Moo et al. 2020), the ubiquity of smartphones—with 69% global use and 6.7 billion subscriptions in 2023—positions these technologies as a strategic infrastructure for democratizing digital support (Statista 2023).

Emerging evidence suggests that mHealth interventions could reduce caregivers' burden and depression by optimizing dementia management and strengthening emotional resilience (Rice et al. 2022; Hepburn et al. 2022). One of their central contributions lies in self‐monitoring, an essential mechanism for health self‐regulation (Kassavou et al. 2022). These tools facilitate real‐time tracking of indicators such as stress, physical activity, and emotional well‐being, fostering immediate awareness of the caregivers' state. Furthermore, they have the potential to assist in goal setting and provide of feedback to facilitate proactive behavioral adjustments (Degroote et al. 2021). Within the context of dementia, these tools integrate monitoring of caregivers' burden, depression, and patient‐related variables (e.g., medication adherence), enhancing reflective capacity and resilience (Rathnayake et al. 2019; Morgan et al. 2022).

### 1.4 Gaps in mHealth Research and Rationale for the Present Review

Although mHealth technologies show substantial promise, current evidence reveals notable gaps. Most studies focus on usability, general features, or acceptability (Chelberg et al. 2022; Ruggiano et al. 2021; Ye et al. 2023), while fewer examine interventions explicitly targeting caregivers' burden and wellbeing, or incorporating monitoring processes. Long‐term effects on caregivers' quality of life and burden also remain insufficiently evaluated. For example, Shin et al. (2022) reported improvements in caregivers' quality of life and competence, yet findings on burden, stress, and depression were inconclusive due to heterogeneity in intervention content, study design, and measurement. Research has further focused on caregivers supporting individuals with dementia in residential care, with limited attention to those providing home‐based care (Costanzo et al. 2020; Daly Lynn et al. 2019; Sohn et al. 2023). Similarly, Kim et al. (2021) highlighted the scarcity of mHealth applications specifically designed to improve caregivers' wellbeing and reduce burden, and noted that little is known about how these tools enhance caregivers' autonomy in decision‐making and health management. Although monitoring has been identified as a key mHealth component (Rathnayake et al. 2019), prior reviews did not distinguish outcomes based on specific intervention features, limiting understanding of how self‐monitoring contributes to reduced burden or improved mental health.

These gaps underscore the need for rigorous, large‐scale evaluations of how mHealth interventions integrate monitoring, enhance caregivers' awareness of their health, and support sustained improvements in burden and wellbeing. Addressing these limitations, the present systematic review examines recent mHealth interventions for family caregivers of people with dementia, focusing on how self‐monitoring supports physical and mental wellbeing. It further evaluates the impact on caregivers' burden, stress, and resilience, offering insights to inform the design of more effective, accessible, and sustainable mHealth solutions that empower caregivers and promote long‐term quality of life.

## 2 Objectives

Our Objective Was to Identify and Analyze mHealth Interventions for Family Caregivers of People With Dementia, Examining Their Potential to Enhance Monitoring and Wellbeing Over the Past 5 Years, With Particular Emphasis on Embedded Monitoring Functions. Our specific objectives were three:

1. To characterize mHealth app features: describe the bibliographic, methodological, and technological characteristics of these apps, including intervention models, variables, measurement tools, and usability aspects linked to self‐management outcomes.
2. To determine whether mHealth interventions reported in the last years include mechanisms that promote self‐regulation, particularly the monitoring component, and to describe how these mechanisms are implemented.
3. To evaluate whether the effects of mHealth interventions on caregiver and caregiving outcomes differ depending on their integration of monitoring components.

## 3 Method

The review was conducted in accordance with the PRISMA 2020 guidelines (Preferred Reporting Items for Systematic Reviews and Meta‐Analyses) for systematic reviews and meta‐analyses, Page et al. (2021).

### 3.1 Information Sources and Search Strategy

Prior to defining the search strategy, a preliminary analysis of the scientific literature was conducted to determine whether similar studies had already been carried out. For this purpose, the Database of Abstracts of Reviews of Effects (DARE) from The Cochrane Library was consulted given its relevance in the field of health intervention reviews. Subsequently, a systematic search of seven electronic databases—MEDLINE, PsycInfo, Psychology & Behavioral Sciences Collection, Psicodoc, and CINAHL via EBSCOhost, Web of Science, and Scopus—was conducted during November 2025. The search strategy used the following Boolean string in the abstract field: (dementia AND caregiver) AND (mHealth OR app OR “mobile application”). These general terms were intentionally selected to ensure an inclusive and comprehensive search, capturing a wide range of studies related to digital health tools for caregivers of individuals with dementia. Searches were limited to English‐language publications between 2019 and November 2025, resulting in a total of 1161 records.

### 3.2 Eligibility Criteria, Study Selection, and Data Extraction Process

Studies were included if they met all six inclusion criteria outlined in Table 1 and excluded if they met any of the eight exclusion criteria. Eligible studies were full‐text articles published in English between 2019 and November 2025, a period marked by significant advances in mHealth technologies. Within this timeframe, we specifically examined the use of mHealth applications designed to support caregivers of people with dementia.

**TABLE 1:** Studies' inclusion and exclusion criteria.

1.	The interventions were aimed at informal (relative) caregivers of people with dementia, who live at home (non‐institutionalized)
2.	The interventions used mobile applications (Apps) on smartphones or tablets
3.	The interventions had at least some of their content available asynchronously (self‐directed)
4.	The studies conducted a pre‐ and post‐intervention assessment
5.	Studies reported the (quantitative) outcomes of an intervention on caregiver psychosocial variables (burden, wellbeing, stress, coping, social support, quality of life, etc.)
6.	Quantitative studies including randomized controlled trials, quasi‐experimental trials, pilot studies, feasibility studies, or preliminary efficacy studies
*Studies' exclusion criteria*	
1.	Interventions targeted healthcare professionals, including nurses, physicians, therapists, clinicians, or professional caregivers.
2.	Interventions targeted individuals without dementia.
3.	Interventions focused on the patient rather than the caregiver.
4.	Interventions utilized computer‐assisted technology, non‐mobile applications, or failed to specify the device used.
5.	Evaluations focused exclusively on usability or engagement, omitting the impact of the app‐based intervention on caregiver well‐being.
6.	Studies focused solely on conceptual applications, design and descriptive processes, or app development without empirical usage evaluation.
7.	Review articles, such as literature or systematic reviews (to ensure the inclusion of primary sources only).
8.	Gray literature and non‐peer‐reviewed formats, including presentations, protocols, commentaries, editorials, abstracts, conference papers, or posters.

*Note:* Six inclusion and eight exclusion criteria are presented; all of the inclusion criteria needed to be met by each study to be included.

Eligibility and inclusion criteria were defined using the PICO framework (Santos et al. 2007): the population (P) comprised family caregivers of people with dementia; the intervention (I) involved mHealth apps; the comparison (C) was omitted as a meta‐analysis was not intended; and the outcomes (O) focused on caregivers' burden and wellbeing. Specifically, the review examined quantitative psychosocial outcomes among family caregivers of people with dementia, focusing primarily on caregivers' burden and wellbeing, as well as related constructs including stress, coping, social support, and quality of life.

**FIGURE 1:**
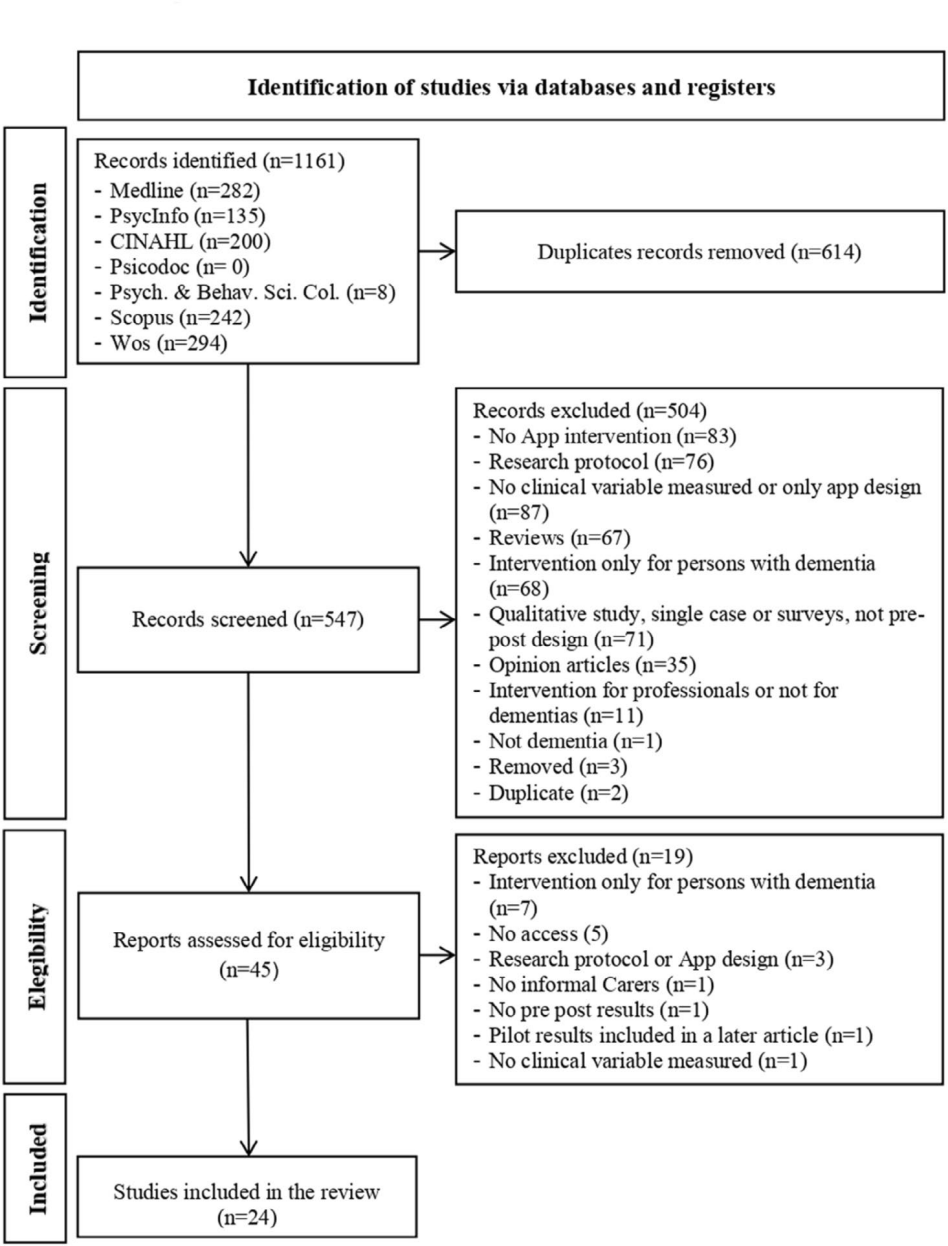
Flow diagram of the article selection process for mHealth app interventions targeting dementia caregivers.

Duplicates from the initial search results were removed using Zotero bibliographic management software. Subsequently, two researchers independently screened titles, abstracts, and full texts in accordance with predetermined inclusion and exclusion criteria. We calculated inter‐rater reliability for 10% of the full sample, resulting in a Cohen's Kappa ranging from 0.66 to 1 for each criterion (see Table S1 and Inter_Rater_Criteria_10 from the Supporting Information), with a total mean of 0.90. The selection process is illustrated in Figure 1 using a PRISMA flow diagram. Of the 1161 intervention studies identified in the search, 24 met all the inclusion criteria. Each of these studies and their implications for mHealth app interventions for family caregivers of people with dementia were thoroughly analyzed.

### 3.3 Evaluating the Risk of Bias in Individual Studies

Given the exploratory nature and methodological heterogeneity of the included studies, a formal methodological quality assessment was conducted only for randomized controlled trials. Specifically, for RCTs (see Figure S1), we applied the Cochrane Risk of Bias 2 (ROB‐2) tool (Sterne et al. 2019). Non‐randomized, pilot, feasibility, and quasi‐experimental studies were descriptively assessed by reporting their key methodological characteristics to support cautious interpretation of the findings.

## 4 Results

The 24 articles included in this review aimed to improve burden and/or wellbeing—whether physical, cognitive, emotional, or social—of family caregivers of people with dementia. However, the approaches used to address these outcomes were highly heterogeneous across studies, as detailed below.

### 4.1 Interventions Outcomes: Caregivers' Burden and Wellbeing

Overall, most studies suggest that mHealth apps may offer benefits for caregivers, although the observed improvements are not always statistically significant and are not consistent across all measured variables, as summarized in Table 2. Interestingly, Smith et al. (2025), notes that higher caregivers' burden is associated with better app perception.

**TABLE 2:** Outcomes related to levels of Active Monitoring Components (AMC).

High AMC			Low AMC		
	ACM	Results		ACM	Results
Goto et al. (2024) Hong et al. (2024)	Regular assessments Needs detection	Significant burden reductions	Park et al. (2020)	Unstructured monitoring	Significant reductions in burden and fatigue
Rodriguez et al. (2023)	Regular assessments	No difference in the patient's neuropsychiatric symptoms			
Goodridge et al. (2021)	EMA	Improvements in emotional wellbeing and emotion‐based coping	Hong et al. (2023)	Unspecific behavior tracking	Improved burden, depression, and life satisfaction
Ruggiano et al. (2024)	Chatbot‐based interaction Self‐assessments	Non‐significant reduction on depression Slight burden improvement.	Romero‐Mas et al. (2021)	Strong peer support No monitoring	Quality of life improvements
Collins‐Pisano et al. (2024)	Cognitive monitoring tools	Stress reduction	Sikder et al. (2019)	Guided mindfulness techniques Non formal tracking	Improved mood and reduced stress
Iacob et al. (2024)	Dashbord and automated coaching	Significant increases in respite time. Coaching component‐specific effects on anxiety	Nguyen et al. (2025)	Asks about doubts and offers informal self‐ and qualitative evaluation of progress and difficulties	Significant improvements in depression, anxiety, stress, knowledge, burden, and social support, sustained at 3 months
Kagwa et al. (2025)	Personal Diary, chat with professional	No impact on burden or depression			
Thompson et al. (2025)	Calls and diary	Significant reduced distress and increased knowledge	Gallegos et al. (2025)	Mindfulness self‐monitoring	Guided breathing produced significant effects on loneliness and emotional regulation
Coleman et al. (2025)	Automated‐coaching system for planning and assessing respite time	Improved positive aspects of care. Stronger effects in those providing > 80% of care			
Plys et al. (2025)	Guided mindfulness	Significant improvement in knowledge of the technique and in the recognition of positive aspects of caregiving	Smith et al. (2025)	Location tracking	Greater acceptance among those with higher burden

Across the reviewed studies, caregivers' burden emerged as the most commonly evaluated outcome. Six interventions—especially those incorporating active monitoring features such as regular assessments or feedback—demonstrated significant reductions in burden (Goto et al. 2024; Hong, Wang, Yang, et al. 2024, 2023; Nguyen et al. 2025; Park et al. 2020; Ruggiano et al. 2024). Other studies (Coleman et al. 2025; Iacob et al. 2024) report increases in respite and satisfaction with care, outcomes that may be linked to caregivers' perceived burden. Moreover, studies such as the one by Leung et al. (2022) noted a statistically significant but not clinically meaningful increase, possibly reflecting heightened awareness without sufficient coping mechanisms. Finally, six studies (Castillo et al. 2024; Collins‐Pisano et al. 2024; Goodridge et al. 2021; Kagwa et al. 2025; Park et al. 2020; Ruggiano et al. 2024), reported no significant change in burden, although several noted slight improvements.

In terms of emotional and psychological wellbeing, benefits were observed in various domains: fatigue (Park et al. 2020), emotional wellbeing (Goodridge et al. 2021; Watcharasarnsap et al. 2020), life satisfaction (Hong et al. 2023), anxiety (Iacob et al. 2024; Nguyen et al. 2025), mood (Sikder et al. 2019), and positive aspects of caregiving (Coleman et al. 2025). Additionally, improvements in dementia knowledge and stress levels were reported in studies like the ones by Castillo et al. (2024) and Collins‐Pisano et al. (2024); while depressive symptoms significantly declined in four interventions. Only one study (i.e., Hong, Wang, Yang, et al. 2024) showed improvements in care recipient outcomes like behavioral and psychological symptoms of dementia (BPSD) or in the relationship between the care and the care recipient (Watcharasarnsap et al. 2020). No significant improvements were found for sleep, general health, or perceived social support. Still, findings from Romero‐Mas et al. (2021) and Neal et al. (2024), highlighted that caregivers' quality of life can be preserved, especially among older caregivers, and is positively associated with e‐health literacy.

### 4.2 Interventions' Outcomes by Levels of Monitoring Features

Several studies integrated active monitoring components (Table 2), such as ecological momentary assessments (EMA), periodic self‐assessments, needs detection, or tracking tools (Collins‐Pisano et al. 2024; Goodridge et al. 2021; Goto et al. 2024; Hong et al. 2024; Ruggiano et al. 2024). The combination of the findings by Iacob et al. (2024) and Coleman et al. (2025) is noteworthy: while the former reports that automated coaching reduces anxiety and increases respite time, the latter indicates that these effects are stronger among caregivers who provide more than 80% of the total care.

In contrast, studies that did not include active monitoring elements showed more mixed results (Hong et al. 2023; Park et al. 2020; Romero‐Mas et al. 2021; Sikder et al. 2019). Some of them (e.g., Leung et al. 2022; Castillo et al. 2024) showed no significant benefits or even an increase in perceived burden. Neal et al. (2024), with a low monitoring level, showed significant effects, likely because they used a familiar messaging platform rather than an external app.

### 4.3 Features of the mHealth Apps' Interventions

The intervention models across studies demonstrated substantial heterogeneity, though eight main categories emerged, as shown in Table 3. The majority of apps (*n* = 9) were built on psychoeducation frameworks, often enhanced with behavioral symptom management strategies. Other studies incorporated social or peer support, mindfulness‐based therapies, guided breathing, or creative approaches like art therapy. While all interventions delivered content via mobile apps, they differed in presentation, interaction, and follow‐up methods. Most apps offered static multimedia materials and included interpersonal contact, whether through professionals (e.g., care managers, clinicians) or peer networks.

**TABLE 3:** Intervention models of the selected mHealth apps.

Studies	Psycho‐education	SPCD handling	Safety, planning & activities	Social & peer support	Clinical info	Therapy	Assessment	AI
Blackberry et al. (2023)	x			x	x		x	
Castillo et al. (2024)	x							
Coleman et al. (2025); Iacob et al. (2024)			x	x			x	
Collins‐Pisano et al. (2024)	x							
Gallegos et al. (2025)	x					x		
Goodridge et al. (2021)						x		
Goto et al. (2024)	x	x		x				
Hong et al. (2023)	x			x				
Hong et al. (2024)	x	x			x			
Kagwa et al. (2025)	x				x	x		
Leung et al. (2022)				x		x		
Neal et al. (2024)			x					
Nguyen et al. (2025)	x	x		x				
Park et al. (2020)	x	x						
Plys et al. (2025)						x		
Rodriguez et al. (2023)	x	x		x			x	
Romero‐Mas et al. (2021)				x				
Ruggiano et al. (2024)	x	x		x	x		x	x
Sikder et al. (2019)						x		
Smith et al. (2025)			x					
Thompson et al. (2025)	x	x	x					
Watcharasarnsap et al. (2020)							x	
Total	13	7	4	9	4	6	5	1

*Note:* The table outlines the intervention models used in the selected mHealth apps, with crosses identifying the components used in each study. For further details, consult the Table S4.

Notably, one intervention integrated an AI‐based chatbot to provide automated support and feedback (Ruggiano et al. 2024), reflecting a growing interest in scalable, interactive tools. Other interventions specifically aimed to plan and encourage the use of respite in caregiving through structured scheduling (Coleman et al. 2025; Iacob et al. 2024). Finally, in the same direction, an application was designed to identify tools for joint activities (Neal et al. 2024).

Monitoring features—essential for supporting self‐regulation—were present in multiple forms (for further details see Table S4). Ten categories were identified. Remarkably, while these tools are innovative, there is a lack of assessments measuring their individual impact independently of other interventions. The tools categories are:

1. *Progress dashboards and automated coaching*: Platforms for monitoring respite hours and goal attainment, featuring reminders, automated coach and objective‐adjustment guidance (Iacob et al. 2024; Coleman et al. 2025; Plys et al. 2025).
2. *Personal diaries and chats*: Individual logging tools and direct communication channels with professionals (Kagwa et al. 2025).
3. *Ecological Momentary Assessments (EMA)*: Systems designed for real‐time emotional state evaluation (Goodridge et al. 2021).
4. *Periodic health self‐assessments*: Instruments measuring caregivers' burden and general health status (Goto et al. 2024; Ruggiano et al. 2024).
5. *Evolutionary needs monitoring*: Tools for detecting shifts in caregiving requirements (Hong et al. 2024).
6. *Cognitive pattern management*: Specific functions to identify and address negative mental schemas (Collins‐Pisano et al. 2024).
7. *Competency self‐monitoring platforms*: Systems to assess dementia‐related knowledge and skills, including virtual reflection sessions (Blackberry et al. 2023).
8. *Geolocation*: Safe‐zone generation for indirect supervision of the care recipient (Smith et al. 2025).
9. *External monitoring*: use of Google Analytics to monitor app usage (Blackberry et al. 2023) or contact with professionals (Kagwa et al. 2025; Thompson et al. 2025).
10. *Mindfulness*: Interventions focused on emotional monitoring and self‐regulation (Gallegos et al. 2025; Kagwa et al. 2025; Plys et al. 2025; Sikder et al. 2019).

While these elements were not uniformly analyzed as standalone predictors of outcomes, their inclusion aligns with a broader shift toward integrating self‐awareness and adaptive feedback mechanisms into digital caregivers' support tools. These apps differed from others that relied primarily on static content delivery and lacked embedded feedback loops or behavior‐tracking functions.

Fewer studies included external monitoring, goal‐setting, behavior tracking, or habit reminders; core aspects of self‐regulation that merit further integration. Their absence limits the capacity of some apps to support adaptive self‐management.

### 4.4 Methodological Characteristics of Included Studies

The methodological design of the selected studies, including the type of study, duration, control groups, sample size, and participant characteristics (caregivers only or caregiver/person with dementia dyads), is detailed in Table 4. These methodological aspects help contextualize the strength and generalizability of the outcomes and clarify how monitoring components were evaluated across different study designs. Sample sizes ranged from as few as nine caregivers (Collins‐Pisano et al. 2024) to 143 (Coleman et al. 2025). Four studies included both caregivers and care recipients (Goto et al. 2024; Hong et al. 2024; Rodriguez et al. 2023; Watcharasarnsap et al. 2020), while the remaining studies focused exclusively on caregivers. Intervention duration ranged from a week (Watcharasarnsap et al. 2020), to 12 months (Hong et al. 2024; Ruggiano et al. 2024). Across all studies, the majority of participants were women (51, 7%–91%), with mean ages ranging from 40 to 87 years. Most caregivers were spouses or adult children of individuals with dementia.

**TABLE 4:** Methodological characteristics of the selected mHealth app intervention studies.

Authors	Country	Design and durability	Control	Sample
Blackberry et al. (2023)	Australia	RCT 8 month	Usual care (stepped wedge)	IC = 37 (86% female, spouse 38%); ACM = 60, living with care recipient (45%)
Castillo et al. (2024)	United States	RCT 2 weeks	Usual care	IC = 130; CG = 41; EG1 = 45; EG2 = 44
Coleman et al. (2025)	Unites States	RCT 16 weeks	Delayed intervention	IC: 143; CMA: 61.06; 82% female
Collins‐Pisano et al. (2024)	United States	OPP 2 weeks	—	IC = 9; CMA = 67.3 (range 42–87); 67% Female; 44% Spouse
Gallegos et al. (2025)	United States	RCT 2 weeks	Active control	IC: 55; CMA: 65 (range 52–81); 91.7% female
Goodridge et al. (2021)	Canada	OPP 12 weeks	—	IC = 53; CMA = 58 years (91% female; spouses, children, friends, and other family members)
Goto et al. (2024)	Japan	OPP 3 month	—	C&C = 13 (84.6% female); Carers age range 40–(>)80
Hong et al. (2023)	United States	OPP 7 weeks	—	IC = 24; CMA = 60 years (71% female, 67% daughters, and 30% spouses)
Hong et al. (2024)	Taiwan	CC 12 month	usual care	C&C = 111; CG = 54; EG = 57. Follow‐up DCP = 52
Iacob et al. (2024)	United States	RCT 16 weeks	Delayed intervention	IC: 163; CMA: 61.7 (range 20–92); 78.9% female; 68.1% Spouse; 24.1% children
Kagwa et al. (2025)	Sweden	OPP 8 weeks	—	IC: 35; CMA: 69.4; 80% female; 68.6 Spouse
Leung et al. (2022)	China	OPP 8 weeks	—	IC = 28; CMA = 59.71 years (71.4% female, 92.9% married, and 78.6% retired or unemployed)
Neal et al. (2024)	The Netherlands	RCT 3 month	Usual care	C&C: 150; CMA: 65.3; 71% female
Nguyen et al. (2025)	Vietman	RCT 7 weeks	Usual care	CI = 172; CMA; 52; 78% female; 81% Spouse
Park et al. (2020)	South Korea	ECG 4 weeks	Informational manuals	IC = 26; CG = 13; EG = 13 (spouses, sons and daughters‐in‐law)
Plys et al. (2025)	United States	RCT 12 weeks	Active control	IC: 95; CMA: 54; 95% female; 59% adults children; 28% spouses
Rodriguez et al. (2023)	United States	RCT 6 months	ABC usual care	DCP = 53; CG = 27; EG = 26 CMA = 62.9 years (77% female; spouses and children)
Romero‐Mas et al. (2021)	Spain	OPP 10 months	—	CF = 38; EG1 = 19 IC + 1 expert IC; EG2 = 19 CF + 3 professionals; CMA = 56 years (79% female; 79% children and 21% spouses and others)
Ruggiano et al. (2024)	United States	OPP 12 month	—	IC = 21; (85.7% female), age range (43–80 years); wife (50%), daughter (22.7%), living with care recipient (77.3%)
Sikder et al. (2019)	United States	OPP 4 weeks	—	CF = 21; CMA = 66.52 years (71% female)
Smith et al. (2025)	United States	OPP 3 month	—	IC: 41; CMA: 66.13; 78.7% female 66.7% Spouse
Thompson et al. (2025)	Australia	OPP 8 weeks	—	C&C: 17; CMA: 60 (range 31–84); 82% female; 76% Spouse
Watcharasarnsap et al. (2020)	Thailand	ECG 1 week	Usual care	C&C = 60 (40 Bangkok; 20 Chonburi); CG = 30; CMA = 41, Range 18 to > 58; Female = 31. Spouses = 8, Children = 8; Friends = 7; Other = 14.

*Note:* Design: ESM: explanatory sequential mixed methods design. WCR: waitlist‐control randomized design. CC: control case. RCT: randomized controlled trial. ECG: experimental with control group; OPP: One‐group pretest‐posttest quasi‐experimental. CRCT: stepped‐wedge, cluster‐randomized controlled trial. Sample and study population: CG = control group; EG = experimental group; IC = informal caregiver; C&C = caregiver and cared for dyad; CMA = caregiver mean age. ABC = aging brain care, individualized care plan with professional care team (geriatrics, social work, and nursing).

Fifteen of the 24 studies were preliminary efficacy trials that lacked a control group, often using single‐group pre‐post designs (e.g., Goodridge et al. 2021; Goto et al. 2024; Sikder et al. 2019; Kagwa et al. 2025; Smith et al. 2025). For instance, Watcharasarnsap et al. (2020), designed a quasi‐experimental study with an experimental group and a control group without random assignment. Romero‐Mas et al. (2021), used a two‐group randomized design where both groups received different forms of intervention, where the difference between the two was based on whether the sessions were led by experienced caregivers or by a team of professionals. Among the more rigorous trials, Park et al. (2020) employed a non‐equivalent control group, while Hong et al. (2024) used a non‐randomized control case design. Blackberry et al. (2023), used a stepped‐wedge cluster randomized trial with 12 communities. Eights studies (Coleman et al. 2025; Castillo et al. 2024; Gallegos et al. 2025; Iacob et al. 2024; Neal et al. 2024; Nguyen et al. 2025; Plys et al. 2025; Rodriguez et al. 2023), were randomized controlled trials (RCTs), wich compared intervention effects with: (1) usual care (Castillo et al. 2024; Nguyen et al. 2025; Rodriguez et al. 2023); (2) active group (Gallegos et al. 2025; Plys et al. 2025) or, (3) with delayed waitlist (Coleman et al. 2025; Iacob et al. 2024). Risk of bias for these RCTs was assessed using the Cochrane ROB‐2 tool (Sterne et al. 2019) and can be consulted in Figure S1.

### 4.5 Variables and Measuring Instruments

Relevant differences were observed in the variables and measurement scales used across the studies (see Table S2). As summarized in Table 5, the most frequently assessed variable was caregivers' burden (*n* = 13), followed by depressive symptoms (*n* = 8). Social support, behavioral and psychological symptoms of dementia (BPSD), and stress were each assessed in four studies, while general health status was utilized in three.

**TABLE 5:** Synthesis of variables and measuring instruments.

Variables	Studies
Caregiver burden	Castillo et al. (2024); Collins‐Pisano et al. (2024); Blackberry et al. (2023); Goodridge et al. (2021); Goto et al. (2024); Hong et al. (2024, 2023); Kagwa et al. (2025); Leung et al. (2022); Nguyen et al. (2025); Park et al. (2020); Ruggiano et al. (2024); Smith et al. (2025)
Depressive symptoms	Hong et al. (2024, 2023); Kagwa et al. (2025); Leung et al. (2022); Nguyen et al. (2025); Plys et al. (2025); Ruggiano et al. (2024); Sikder et al. (2019)
Social support	Blackberry et al. (2023); Hong et al. (2023); Leung et al. (2022); Nguyen et al. (2025)
BPSD: behavioral and psychological symptoms of dementia	Hong et al. (2024); Park et al. (2020); Rodriguez et al. (2023); Thompson et al. (2025)
Stress	Castillo et al. (2024); Collins‐Pisano et al. (2024); Park et al. (2020); Plys et al. (2025)
General health status	Castillo et al. (2024); Leung et al. (2022); Ruggiano et al. (2024)
Quality of life	Neal et al. (2024); Romero‐Mas et al. (2021)
Relation and psychological wellbeing	Watcharasarnsap et al. (2020)
Respite and anxiety	Coleman et al. (2025); Iacob et al. (2024)
Positives aspects of care	Coleman et al. (2025); Plys et al. (2025)

### 4.6 Usage, Usability, and Acceptance of the mHealth Apps

App usage data were inconsistently reported across studies. Ruggiano et al. (2024) and Blackberry et al. (2023), provided detailed analytics, documenting logon, user actions, visited pages per session, chatbot interaction, or average duration of sessions. While this suggests early interest, sustained interaction appeared limited. This lack of report of systematic engagement, especially regarding monitoring tools such as log‐ins, feedback use, or self‐tracking, represents a notable gap in evaluating how self‐regulation features contribute to long‐term adherence. Smith et al. (2025), report that 31.7% used the geolocation app daily and 89.8% indicated being “extremely” or “somewhat” satisfied. Thompson et al. (2025), report high acceptance and an app use of 3–4 times per week. Similarly, Gallegos et al. (2025), reported that most completed the sessions and described the activity as comfortable and satisfying. Finally, Plys et al. (2025), reported high enrollment and moderate‐to‐high satisfaction and credibility, being used for almost 1 h per week on average.

Usability was assessed in most studies using tools like the SUS and MARS, alongside qualitative feedback. Findings consistently highlighted app accessibility and perceived usefulness (e.g., Collins‐Pisano et al. 2024; Hong et al. 2023; Leung et al. 2022; Thompson et al. 2025), regardless of variability in users' age and computer skills. However, challenges in maintaining user engagement over time were common. For example, Rodriguez et al. (2023) observed a drop in usage after 3 months, and Castillo et al. (2024) noted usability concerns despite participants finding the content informative. Nevertheless, qualitative data underscored the emotional value of mHealth apps—caregivers reported feeling more emotionally supported, calm, and less isolated (Leung et al. 2022; Romero‐Mas et al. 2021; Sikder et al. 2019).

## 5 Discussion

Dementia caregiving places impactful physical, emotional, and financial strain on caregivers, frequently leading to neglect of their own health and wellbeing (Riffin et al. 2019). The global rise in dementia prevalence, coupled with the increasing reliance on family caregivers, underscores the urgent need for scalable and accessible interventions. Amid these challenges, self‐regulation—the ability to monitor, evaluate, and adapt one's behaviors and emotions—has been recognized as a crucial mechanism for promoting caregivers' resilience and wellbeing (Panzeri et al. 2024).

mHealth apps have emerged as a promising solution, offering tailored resources and support to enhance self‐regulation. These tools allow caregivers to access assistance ubiquitously and instantly, effectively overcoming barriers such as geographic location and time constraints (Laver et al. 2020). Despite their potential, existing research has primarily focused on usability and general features, leaving a significant gap in understanding how these tools integrate self‐regulation mechanisms, such as monitoring, and their long‐term impacts on caregivers' burden and wellbeing.

This systematic review aimed to identify and analyze mHealth app interventions for family caregivers of people with dementia, with a particular focus on their potential to enhance monitoring and caregivers' wellbeing as reported over the past 5 years. In the next sections we discuss our results targeting each of our specific objectives: (1) describe the key features and characteristics of mHealth apps for caregivers; (2) examine whether and how mHealth apps include monitoring mechanisms; (3) evaluate whether monitoring components influence the efficacy of mHealth interventions.

### 5.1 Key Features and Characteristics of mHealth Apps for Caregivers

The 24 studies reviewed highlighted the diverse approaches and functionalities of mHealth interventions, reflecting the multifaceted needs of caregivers. The works included showed methodological heterogeneity, with considerable preliminary or non‐controlled designs. Psychoeducation‐based interventions were the most common model. Apps such as “Dementia Talk” and “CLEAR Dementia Care” aimed to enhance caregivers' understanding of dementia and equip them with strategies to address caregiving challenges (Castillo et al. 2024; Park et al. 2020). By increasing caregivers' knowledge and problem‐solving skills, psychoeducation helps caregivers to navigate daily responsibilities with greater confidence, potentially reducing their burden (Frias et al. 2020; Torkamani et al. 2014). Notably, some of these apps included monitoring components, such as behavior tracking tools, which allowed caregivers to observe care recipients' symptoms and adjust their caregiving strategies accordingly.

Social support interventions emphasized fostering community among caregivers. Apps such as “WECARE” and “Estic amb tu [I'm with you]” facilitated peer‐to‐peer networks and group discussions through chat features and moderated forums (Hong et al. 2023; Romero‐Mas et al. 2021). Social connections alleviate caregivers' isolation and loneliness, offering emotional reinforcement. These apps also integrated monitoring features, such as self‐assessment tools, enabling caregivers to track their emotional states and discuss their experiences with peers or professionals, further reinforcing social support.

Regarding mindfulness‐based psychotherapy interventions, apps like “Ethica” and the “MIT App” included mindfulness exercises to enhance emotional regulation and reduce stress (Goodridge et al. 2021; Sikder et al. 2019). Mindfulness practices, supported by monitoring mechanisms such as reflective prompts and reminders, encouraged caregivers to recognize and regulate their emotional states, fostering resilience (Liu et al. 2018; van Boekel et al. 2019).

The content delivery methods also varied significantly across interventions. Apps such as “Dementia Talk”, “Brain Care Notes”, and “Ethica” provided permanent access to educational materials, while others, like “WECARE,” incorporated interactive features such as live group meetings. “Brain Care Notes” stood out by offering direct access to care advisors, enabling caregivers to receive personalized guidance based on the results of a self‐assessment (Rodriguez et al. 2023). Such heterogeneity reflects the flexibility and adaptability of mHealth apps to cater to diverse caregivers' needs.

While these features show promise, the variations in measured variables reflect diverse conceptualizations of caregivers' wellbeing underlining the need for greater standardization. Moreover, maintaining caregivers' engagement remains a challenge. Several studies observed declines in app usage over time, even when financial incentives were offered (Castillo et al. 2024; Rodriguez et al. 2023). This highlights the need for engaging user‐friendly designs and features that seamlessly integrate into caregivers' daily routines, while minimizing manual data entry or answer questionnaires, such as EMAs (ecological momentary assessment) tools. Furthermore, variability in e‐health literacy underscores the importance of designing apps that are accessible to diverse caregiver populations (Goodridge et al. 2021; Romero‐Mas et al. 2021).

Addressing these barriers is crucial to ensure that mHealth interventions not only offer immediate support but also sustain long‐term benefits. Central to this goal is the integration of self‐regulation mechanisms—particularly monitoring, which has emerged as a pivotal feature in empowering caregivers to proactively manage their emotional states, caregiving strategies, and overall well‐being. The importance of incorporating such mechanisms is explored in the following section with more depth.

### 5.2 Self‐Regulation Enhanced Mechanisms by mHealth Apps: The Role of Monitoring

Monitoring, a key component of self‐regulation, was utilized in diverse ways across the reviewed interventions, demonstrating its potential to support caregivers' wellbeing. Apps like “Dementia Talk” and “CLEAR Dementia Care” incorporated behavior tracking tools, enabling caregivers to log and evaluate care recipients' symptoms, such as behavioral disturbances, and adapt their caregiving strategies in real time (Castillo et al. 2024). Similarly, “Brain Care Notes” provided personalized care tips based on weekly assessments, empowering caregivers with actionable insights to enhance caregiving efficacy and reduce stress (Rodriguez et al. 2023).

Monitoring also played a central role in stress management and emotional regulation. For instance, the “MIT App” used automated daily reminders to encourage mindfulness practices, helping caregivers reflect on their emotional states and adopt stress‐reduction techniques consistently (Sikder et al. 2019). Likewise, “Ethica” integrated momentary ecological assessments, prompting caregivers to self‐report their emotional states daily and fostering emotional awareness and resilience (Goodridge et al. 2021).

Apps promoting social support also leveraged monitoring to strengthen caregivers' engagement. “WECARE” included self‐assessment tools that caregivers could use to track their stress levels and share insights during group discussions, creating a supportive community dynamic (Hong et al. 2023). Similarly, “E‐Painting” encouraged caregivers to reflect on their emotions through thematic art exercises, providing a creative form of self‐monitoring (Leung et al. 2022).

Monitoring as a foundational component of self‐regulation plays a crucial role in initiating and sustaining the self‐regulation process. By enabling caregivers to observe and track their own emotional states, caregiving behaviors, and care recipients' symptoms, monitoring provides the essential awareness needed to identify stressors, evaluate the effectiveness of caregiving strategies, and recognize areas for improvement (Schulman‐Green et al. 2012; Schulz and Sherwood 2008).

Awareness activates subsequent self‐regulation strategies, such as goal‐setting, problem‐solving, and adaptive responses, which empower caregivers to address challenges more effectively (Schulman‐Green et al. 2012). For example, behavior tracking tools in apps like “Dementia Talk” and personalized assessments in “Brain Care Notes” not only enhance caregivers' understanding of their caregiving dynamics but also enable them to implement targeted strategies to alleviate stress and improve caregiving practices.

As caregivers consistently engage with monitoring features, such as mindfulness reminders or self‐assessment tools, they develop greater emotional resilience and problem‐solving capacity (Goodridge et al. 2021; Sikder et al. 2019). This process not only reduces caregiving burden by improving coping mechanisms and care strategies, but also fosters emotional wellbeing by mitigating stress and promoting a sense of control and empowerment in their caregiving roles (Pinquart and Sörensen 2003; Schulz and Sherwood 2008).

These findings support the notion that beyond functionality, mHealth apps have potential to foster emotional wellbeing. Nevertheless, despite these promising implementations, inconsistencies in the use and evaluation of monitoring mechanisms were evident. Interventions incorporating more structured or active monitoring components tended to report more consistent improvements across psychosocial outcomes; however, these effects were not uniform and varied depending on intervention design, intensity, and caregivers' characteristics (e.g., Kagwa et al. 2025; Park et al. 2020; Ruggiano et al. 2024). Additionally, the lack of standardized metrics for evaluating monitoring effectiveness complicates efforts to assess its impact on caregivers' burden and wellbeing comprehensively. The lack of consistent monitoring‐related engagement data (e.g., log‐ins, tracking behavior, and feedback loops) across studies highlights a critical gap in understanding how self‐regulation—especially through monitoring—drives sustained impact.

In response to this variability, and to support a more structured interpretation of monitoring features across interventions, we derived a descriptive framework to categorize the degree of monitoring incorporated in mHealth apps for caregivers. This framework, presented in Supporting Information 3, is based on patterns observed across the included studies. Importantly, this framework was not applied as a formal evaluative tool but is proposed as a conceptual aid to guide future research and intervention design.

These inconsistencies underscore the importance of understanding not only how monitoring mechanisms are implemented but also their influence on the overall efficacy of mHealth interventions. We discuss it in the next subsection.

### 5.3 Effects of Monitoring Mechanisms on mHealth Apps Efficacy

Regarding comparisons between interventions incorporating monitoring constructs and to those not incorporating them, interventions incorporating robust or intensive monitoring mechanisms—and, therefor, activating the full self‐regulation cycle—yielded greater improvements in caregivers' outcomes (e.g., Goodridge et al. 2021; Hong et al. 2023; Park et al. 2020; Sikder et al. 2019). These features allowed caregivers to recognize stressors, evaluate their strategies, and adapt effectively, leading to enhanced wellbeing and caregiving efficacy. Conversely, apps with limited or absent monitoring features showed inconsistent outcomes. For instance, interventions more focused on education than on monitoring and feedback (Castillo et al. 2024), or those primarily oriented toward expression and social contact (Leung et al. 2022), were less effective in reducing caregivers' burden than those that, in addition to providing information and supervision, promote monitoring and a subsequent social support along with coping strategies. Then, the role of monitoring in engaging the self‐regulation process underscore its value as a key design element for improving caregivers' outcomes.

Mindfulness‐based interventions demonstrated the strongest effects on emotional wellbeing, with apps like “Ethica” and the “MIT App” promoting stress reduction through reflective practices (Goodridge et al. 2021; Sikder et al. 2019). Monitoring mechanisms, such as daily reminders and reflective prompts, sustained engagement with these practices and enhanced their efficacy. Interventions incorporating multiple elements of the self‐regulation cycle—particularly monitoring combined with feedback or coaching—were more likely to report improvements in caregivers' burden or wellbeing (see also the meta‐analysis by Saragih et al. (2024), which found that mindfulness‐based interventions incorporating self‐regulation components effectively improved mental health outcomes in caregivers).

The integration of monitoring with social support further reinforced app efficacy. For example, “WECARE” combined self‐assessment tools with moderated group discussions, fostering peer feedback and community support (Hong et al. 2023). This approach reduced caregivers' isolation and empowered participants to evaluate their wellbeing collectively. Similarly, apps like “Estic amb tu ‐ Estoy Contigo” created opportunities for shared reflection, building both emotional resilience and a sense of community (Romero‐Mas et al. 2021). Therefore, robust monitoring mechanisms amplify the benefits of other intervention components, such as psychoeducation and social support.

Recent studies provide further nuances regarding the role of specific monitoring‐related components. Evidence from Iacob et al. (2024) suggests that automated coaching features may have a stronger impact on caregivers' anxiety than passive dashboard‐based monitoring alone. Complementary findings from Coleman et al. (2025), which evaluated the same intervention using different outcome variables, indicate that these effects were particularly pronounced among caregivers providing more than 80% of the total care. Taken together, these findings suggest a differential effect of monitoring mechanisms depending on both the type of component implemented and the caregiver's level of care involvement: a more active and personalized support is especially beneficial for caregivers with greater needs.

When comparing our results to the broader mHealth review by Agarwal et al. (2021) focused on chronic disease management, we find similarities in the role of monitoring. Both reviews underscore that comprehensive monitoring tools are associated with better adherence and outcomes. However, the chronic disease studies often utilized quantitative real‐time tracking (e.g., glucose levels), whereas our dementia caregivers' studies primarily used reflective and self‐reported monitoring, such as emotional state tracking or care recipient symptom logs.

Additionally, when compared with the findings from Rathnayake et al. (2019), which reviewed mHealth apps as educational and supportive resources for dementia caregivers, our findings extend this discussion about psychoeducation utility by highlighting the critical role of monitoring in enhancing intervention efficacy. Rathnayake et al. (2019) identified education as a key feature in mHealth apps, often supplemented by links to external resources or supportive tools, yet did not emphasize monitoring as a mechanism for improving outcomes. In contrast, our results suggest that monitoring adds an essential layer to mHealth interventions by facilitating self‐regulation, enabling caregivers to make real‐time adjustments to their caregiving strategies, and sustaining emotional wellbeing through reflective practices.

Despite these benefits, sustaining engagement with monitoring tools remains a challenge. Declines in app usage over time, even in interventions with robust monitoring, suggest the need for dynamic, user‐centered designs that adapt to caregivers' evolving needs (Castillo et al. 2024; Rodriguez et al. 2023). Achieving long‐term efficacy requires not only robust monitoring but also adaptive and engaging intervention designs. Future research should focus on standardizing monitoring metrics and developing personalized, flexible interventions to ensure sustained caregivers' engagement and maximized impact.

Importantly, some interventions with minimal or no formal monitoring components also demonstrated beneficial outcomes, particularly when delivered through familiar platforms or when emphasizing strong social or informational support (e.g., Neal et al. 2024; Romero‐Mas et al. 2021). This suggests that monitoring is not a necessary condition for effectiveness but may enhance or structure self‐regulation processes when appropriately integrated. Additionally, the relatively short duration of follow‐up across most studies limits conclusions regarding the sustainability of intervention effects. While several interventions reported short‐term improvements in caregivers' burden or wellbeing, it remains unclear whether these benefits persist over time or require continued engagement with monitoring and feedback features. Understanding the long‐term dynamics of self‐regulation processes in mHealth interventions represents an important direction for future research.

### 5.4 Practical Implications

mHealth apps represent a transformative and scalable solution to the challenges faced by dementia caregivers, offering flexible, immediate access to resources that improve caregiving knowledge, emotional resilience, and social support. These interventions address critical gaps in traditional caregiving resources by overcoming barriers related to time, geography, and the availability of professional services. Through the integration of self‐regulation mechanisms, particularly monitoring, mHealth apps empower caregivers to adopt a proactive approach to managing both their wellbeing and caregiving responsibilities. Monitoring tools allow caregivers to gain real‐time insights into their emotional states, caregiving strategies, and care recipients' needs, fostering adaptive responses and enhancing caregiving efficacy (e.g., Castillo et al. 2024; Rodriguez et al. 2023). The marked variability in both the presence and operationalization of monitoring components across the 24 studies included further underscores the need for a shared conceptual framework to describe and compare monitoring intensity in mHealth interventions.

However, the success of these interventions depends on their ability to sustain caregivers' engagement and ensure accessibility across diverse user populations. Engagement often diminishes over time, highlighting the importance of designing apps that are intuitive, interactive, and seamlessly integrated into caregivers' daily routines. Dynamic monitoring features that evolve with the changing needs of caregivers and personalized content tailored to the caregiver's role (Goodridge et al. 2021), health literacy (Romero‐Mas et al. 2021), and cultural context (Hong et al. 2023) are crucial to maximize the utility of these apps. Moreover, integrating peer support and professional guidance within app platforms can enhance caregivers' sense of community and reinforce their confidence in applying caregiving strategies. In this sense, some publication support the fact that ‘human presence’ alongside the app enhances its effectiveness (Andersson 2018).

The implications of these advancements extend beyond individual caregivers and their families. By reducing caregivers' burden, enhancing emotional resilience, and improving quality of life, mHealth apps contribute to the sustainability of healthcare systems. Improved caregivers' wellbeing can delay care recipient institutionalization, reduce hospital admissions, and mitigate the economic strain on healthcare providers (Castillo et al. 2024; Rodriguez et al. 2023; Romero‐Mas et al. 2021). Furthermore, empowering caregivers with effective monitoring tools aligns with public health priorities of promoting autonomy, reducing mental health disparities, and enhancing the continuity of care for people with dementia (Goodridge et al. 2021). But, as a first step, it is imperative to facilitate the training of professionals in the utilization of Apps for social and healthcare purposes, in addition to the regulation thereof (Martí‐Noguera and Soto‐Pérez 2023).

For policymakers, these findings underscore the need to support the development, evaluation, and dissemination of mHealth technologies. Public funding, collaborative research, and industry partnerships can help ensure that these tools are accessible, evidence‐based, and culturally inclusive. Encouraging caregivers' use of mHealth apps through educational campaigns and e‐health literacy programs will be equally critical to achieving widespread adoption and impact.

In conclusion, mHealth apps hold immense potential to revolutionize caregiving for people with dementia by addressing caregivers' complex needs through tailored, scalable, and flexible solutions. Continued investment in innovative, user‐centered designs and rigorous research will be essential to harnessing their full potential, benefiting not only caregivers and care recipients but also the broader healthcare ecosystem.

### 5.5 Limitations and Future Research

This systematic review highlights important insights into the role of mHealth apps in supporting caregivers of people with dementia, but it also has notable limitations that must be considered when interpreting the findings. First, the review relied on a systematic approach rather than a meta‐analysis, limiting the ability to statistically compare outcomes across studies. This was compounded by the heterogeneity of intervention designs, outcome measures, and methodologies, which made it challenging to draw definitive conclusions about the efficacy of mHealth apps.

Second, the sample sizes across the included studies were generally small, reducing the statistical power to detect significant differences or generalize findings to broader caregivers populations. The heterogeneity across the 24 studies meeting the inclusion criteria further restricts the representativeness of the results and highlights the need for more research in this field. In addition, although monitoring features were a central focus of this review, most studies did not isolate their independent contribution to outcomes, limiting causal inferences regarding their specific role within multicomponent interventions.

Third, only articles published in English and reporting quantitative results were included, resulting in a language bias and excluding potentially relevant qualitative research that could provide valuable insights into caregivers' lived experiences with mHealth interventions.

Fourth, the observed heterogeneity in intervention designs and outcomes also underscores the challenges in standardizing the evaluation of mHealth apps. The lack of consistent metrics for assessing monitoring mechanisms, caregiver burden, time spent on care, other services that support care, or emotional wellbeing limits the ability to compare results and draw robust conclusions about the effectiveness of specific app features.

Despite these limitations, the findings of this review are encouraging and highlight the increasing potential of mHealth apps to provide continuous, remote, and immediate support to caregivers of people with dementia. However, future research should address these gaps by conducting large‐scale, randomized controlled trials with more diverse caregiver populations. Comparative studies analyzing the relative effectiveness of different intervention models and app features, such as monitoring and multicomponent content delivery, would be particularly valuable. Additionally, integrating real‐time data collection tools, such as momentary ecological assessments (EMA), could allow for personalized and adaptive interventions tailored to the unique needs of caregivers.

Finally, future investigations should consider the role of e‐health literacy, caregiver profiles (e.g., age, relationship to the care recipient), and the stage of the disease in determining the usability and effectiveness of mHealth apps. Exploring the feasibility of delivering multicomponent interventions via platforms already familiar to caregivers, such as instant messaging or social media apps, could further reduce technological barriers and enhance engagement. By addressing these limitations, future research can contribute to the development of more comprehensive, accessible, and effective mHealth solutions for caregivers of people with dementia.

## 6 Conclusions

Dementia caregiving imposes substantial physical, emotional, and financial burdens, underscoring the urgent need for scalable, accessible solutions. mHealth apps have emerged as promising tools, offering flexible, immediate support through features that enhance self‐regulation, particularly monitoring. By allowing caregivers to track emotional states, caregiving strategies, and care recipients' symptoms, these apps empower them to adapt their approaches and manage stress effectively. The reviewed studies revealed diverse intervention models, including psychoeducation, social support, and mindfulness, where apps incorporating robust monitoring mechanisms demonstrated greater efficacy in reducing caregiver burden and enhancing emotional well‐being. Features such as behavior tracking, reflective prompts, and peer‐to‐peer support reinforced monitoring, enabling caregivers to identify stressors, evaluate strategies, and respond adaptively.

However, challenges remain in sustaining engagement and ensuring accessibility for caregivers with varying levels of e‐health literacy. While monitoring mechanisms proved to be instrumental in activating self‐regulation processes, the decline in app usage over time suggests the need for dynamic, personalized designs that evolve with caregivers' changing needs. Standardizing monitoring metrics and expanding evaluations through larger, more diverse studies will be critical to validating their long‐term impact. Despite these limitations, mHealth apps hold immense potential to transform dementia caregiving, not only improving caregivers' quality of life but also reducing systemic healthcare burdens and ensuring better outcomes for care recipients. With continued innovation and targeted research, these tools can revolutionize caregiver support and bridge critical gaps in dementia care.

## Funding

The authors have nothing to report.

## Conflicts of Interest

The authors declare no conflicts of interest.

## Supporting information


**Figure S1:** Traffic Light Risk of Bias Using ROB‐2.
**Table S1:** Studies' Exclusion Criteria and Corresponding Cohen's Kappa Inter‐rater Reliability.
**Table S2:** Intervention Variables and Measurement Scales.
**Table S3:** Guide to Determine the Level of Monitoring in an Intervention.
**Table S4:** Features of the Selected mHealth Apps: Content, Monitoring and Outcomes.

## Data Availability

Supporting Information is available for consultation via the following link, and on request from the corresponding author. https://osf.io/kjqm4/?view_only=7621a2df85e14edbbcf8dcc0c6c74f5d.
