# Supplementary material for: mHealth To Promote Monitoring and Self‐Regulation Among Caregivers of People With Dementia: A Systematic Review
Source: Psych J. 2026 Apr 5;15(2):e70092. doi: 10.1002/pchj.70092 (PMC13052052; doi:10.1002/pchj.70092)
Supplement: Supplementary file 1 — Figure S1: Traffic Light Risk of Bias Using ROB‐2. Table S1: Studies' Exclusion Criteria and Corresponding Cohen's Kappa Inter‐rater Reliability. Table S2: Intervention Variables and Measurement Scales. Table S3: Guide to Determine the Level of Monitoring in an Intervention. Table S4: Features of the Selected mHealth Apps: Content, Monitoring and Outcomes. [file PCHJ-15-e70092-s001.zip › Figure S1.pdf]

Figure S1

Traffic Light Risk of Bias Using ROB-2

		D1 Randomization	D2 Deviations	D3 Missing data	D4 Outcome measurement	D5 Selection of reported result	Overall risk
Study	Rodríguez et al. 2023	+	+	-	+	-	-
	Castillo et al. (2023)	+	-	X	-	-	X
	Blackberry et al. (2023)	+	X	X	-	+	X
	Iacob et al. (2024)	+	-	+	-	+	-
	Neal et al. (2024)	+	-	-	-	+	-
	Nguyen et al. (2025)	+	+	+	-	+	-
	Coleman et al. (2025)	-	-	+	-	+	-
	Gallegos et al. (2025)	+	+	+	-	+	-
	Plys et al., (2025)	+	-	-	-	+	-
