# Supplementary material for: mHealth To Promote Monitoring and Self‐Regulation Among Caregivers of People With Dementia: A Systematic Review
Source: Psych J. 2026 Apr 5;15(2):e70092. doi: 10.1002/pchj.70092 (PMC13052052; doi:10.1002/pchj.70092)
Supplement: Supplementary file 1 — Figure S1: Traffic Light Risk of Bias Using ROB‐2. Table S1: Studies' Exclusion Criteria and Corresponding Cohen's Kappa Inter‐rater Reliability. Table S2: Intervention Variables and Measurement Scales. Table S3: Guide to Determine the Level of Monitoring in an Intervention. Table S4: Features of the Selected mHealth Apps: Content, Monitoring and Outcomes. [file PCHJ-15-e70092-s001.zip › Table S1.pdf]

Table S1

Studies' Exclusion Criteria and Corresponding Cohen's Kappa Inter-rater Reliability

number	Description	Examples of Excluded Studies	Inter-rater reliability *
I	The interventions were aimed at health professionals, such as nurses, doctors, therapists, clinicians, professional caregivers or at people without dementia	Huang et al. (2024)	.85
II	Interventions were aimed at the patient rather than the caregiver	De-Rosende-Celeiro et al. (2024)	.79
III	Interventions were based on computer-assisted technology or non-mobile applications or did not specify the device	DuBose et al. (2024)	.66
IV	The evaluations were only about usability or participation and not about the effects of the App intervention on caregivers' wellbeing	Smith. (2025).	.93
V	The studies focused only on a conceptual application, the description and design process, the development of an application without evaluation of use, or used another type of design (e.g., qualitative, survey)	Munk et al. (2025).	.96
VI	The studies were review-based, such as literature reviews or systematic reviews (to ensure that only primary sources of information were included).	de-Moraes-Ribeiro et al. (2024)	1
VII	The studies were presentations, protocols, comments, opinions, abstracts, conference papers or posters.	Cho et al. (2024)	1
VIII	The study was focused on bio-physiological models testing, non-human participants.	Devara et al. (2025)	1

Note. * Cohen's Kappa

References

Cho, E., Yang, M., Kim, M. J., Hwang, S., Kim, E., & Cho, J. (2024). Effectiveness of a mobile app-based individualized non-pharmacological intervention on behavioral and psychological symptoms of dementia in community-dwelling older adults: Study protocol for a randomized control trial. *Journal Of Korean Gerontological Nursing*, *26*(3), 248-256. https://doi.org/10.17079/jkgn.2024.00521

de-Moraes-Ribeiro, F. E., Moreno-Cámara, S., Da-Silva-Domingues, H., Palomino-Moral, P. Á., & Del-Pino-Casado, R. (2024). Effectiveness of Internet-Based or Mobile App Interventions for Family Caregivers of Older Adults with Dementia: A Systematic Review. *Healthcare*, *12*(15), 1494. https://doi.org/10.3390/healthcare12151494

De-Rosende-Celeiro, I., Francisco-Gilmartín, V., Bautista-Blasco, S., & Ávila-álvarez, A. (2024). Co-creation and evaluation of an app to support reminiscence therapy interventions for older people with dementia. *Digital Health*, *10*. https://doi.org/10.1177/20552076241261849

Devara, D., Sharma, B., Goyal, G., Rodarte, D., Kulkarni, A., Tinu, N., Pai, A., & Kumar, S. (2025). MiRNA-501-3p and MiRNA-502-3p: A Promising Biomarker Panel for Alzheimer's Disease. *bioRxiv (Cold Spring Harbor Laboratory)*. https://doi.org/10.1101/2025.01.09.632227

DuBose, L., Fan, Q., Fisher, L., Hoang, M., Salha, D., Lee, S., Ory, M. G., & Falohun, T. (2024). Development and Evaluation of a Web-Based Platform for Personalized Educational and Professional Assistance for Dementia Caregivers: Proposal for a Mixed Methods Study. *JMIR Research Protocols*, *13*, e64127. https://doi.org/10.2196/64127

Huang, H., Chao, Y., Kuo, C., Sung, Y., Shyu, Y. L., & Hsu, W. (2024). Development of a Dementia Case Management Information System App: Mixed Methods Study. *JMIR Aging*, *7*, e56549. https://doi.org/10.2196/56549

Munk, S., Toohey, R., Remtilla, A., D'Cunha, N. M., Gibson, D., Isbel, S., Smyth, A., & Bail, K. (2025). "I forgot she used to make chocolate cake": Digital storytelling supporting person-focussed dementia care: A qualitative thematic analysis. *Dementia*. https://doi.org/10.1177/14713012251317761

Smith, M. L., Wilson, A. D., Knebl, J., Hilsabeck, R. C., Reuter, K., Aguirre, A., Harty, B., Kew, C. L., Lee, S., & Ory, M. G. (2024). Utilization and perceived usefulness of monitoring technology for family caregivers of people living with Alzheimer's disease and related dementias. *Journal Of Alzheimer S Disease*. https://doi.org/10.1177/13872877241300078
