# Supplementary material for: mHealth To Promote Monitoring and Self‐Regulation Among Caregivers of People With Dementia: A Systematic Review
Source: Psych J. 2026 Apr 5;15(2):e70092. doi: 10.1002/pchj.70092 (PMC13052052; doi:10.1002/pchj.70092)
Supplement: Supplementary file 1 — Figure S1: Traffic Light Risk of Bias Using ROB‐2. Table S1: Studies' Exclusion Criteria and Corresponding Cohen's Kappa Inter‐rater Reliability. Table S2: Intervention Variables and Measurement Scales. Table S3: Guide to Determine the Level of Monitoring in an Intervention. Table S4: Features of the Selected mHealth Apps: Content, Monitoring and Outcomes. [file PCHJ-15-e70092-s001.zip › Table S2.pdf]

Table S2

Intervention Variables and Measurement Scales

Authors and Year	Intervention Variables	Measurement Scales
Blackberry et al. (2023)	Burden, Social Support, Engagement, cost	ZBI<br>MOS-SSS
Castillo et al. (2023)	Knowledge of dementia; bless you; stress; caregiver burden; usability and app quality	DKAT2; SF-12; PSS-10; ZBI-22; SUS;<br>MARS-SQS; semi-structured interviews
Coleman et al. (2025)	Positive Aspects of Caregiving, memory and behavioral problems, time for care	PAC, MBPC
Collins-Pisano et al. (2024)	Caregiver: burden, strain and stress	ZBISF-12; MCSI; CSAQ
Gallegos et al. (2025)	Loneliness, emotion regulation, self-compassion; practice and adherence to the intervention, acceptability	UCLA-LS; DERS; SCS
Goodridge et al. (2021)	Caregiver burden; coping styles; emotional wellbeing; "How do you feel today?"; App Utility & Usability	BSFC; Brief-COPE; WHO-5; EMAs; fields notes and interview
Goto et al. (2024)	Behavioural and psychological symptoms of dementia, caregiver Burden	ABS, J-ZBI-22, interview
Hong et al. (2023)	Depressive symptoms; caregiver burden; life satisfaction; social support; App utility	CES-D; ZBISF-12; SWLS; SSS
Hong et al. 2024)	Burden, depression, severity of behavioral and psychiatric symptoms, needs	CDR, NPI, CES-D; ZBI-22; DCQI; IFCN
Iacob et al. (2024)	Respite, anxiety	Respite; PROMIS
Kagwa et al. (2025)	Burden, depression	ZBISF-12, PHQ-9

Table S2

Intervention Variables and Measurement Scales

Authors and Year	Intervention Variables	Measurement Scales
Leung et al. (2022)	Caregiver burden; depressive symptoms; bless you; social support; Usability App	ZBISF-12; PHQ-9; SRH; mMOS-SS; Focus group and phone interview
Neal et al. (2024)	Health quality of life, sense of competence, cost	EQ-5D-5L, SSCQ
Nguyen et al. (2025)	Depression, anxiety, stress, burden, dementia knowledge, social support	DASS-21; NIL&TS; ZBI-4; MSPSS
Park et al. (2020)	Stress; fatigue; dream; caregiver burden; SPCD	Cortisol in saliva; Piper Fatigue Scale; Lee's Sleep Efficiency Scale; ZBI-22; NPI
Plys et al., (2025)	Acceptability, adherence, mindfulness process, stress, anxiety, depression	CSQ-3; CEQ; AMPS; CRS-PG; PSS-10; HADS-A; HADS-D
Rodriguez et al. (2023)	SPCD; caregiver burden; App usability; App acceptance	NPI; NPI-CD; SUS; BIQ
Romero-Mas et al. (2021)	Quality of life; functional impairment of the person with dementia; eHealth literacy; App utility	WHOQOL-BREF, Barthel's Index; eHEALS; two open-ended question about experience.
Ruggiano et al. (2024)	Communication with providers, depression, health literacy about Alzheimer disease, caregiver burden, user data	MCCWP-3; PHQ-4; ADKS; ZBI-22; CareHeroes anonymized user data
Sikder et al. (2019)	Depressive symptoms; mood; Care Experience	MARS; QIDS; PANAS; interview
Smith et al. (2025)	Burden, App use and satisfaction	ZBISF-12
Thompson et al. (2025)	Acceptability, usability, symptoms, knowledge about music use	AES, SUS, NPI, CMAI, KAMU

Table S2

Intervention Variables and Measurement Scales

Authors and Year	Intervention Variables	Measurement Scales
Watcharasarnsap et al. (2020)	Relation; Psychological wellbeing	Ah-C-PwDRQ<br>PWBQ

Note. *CSQ*-3: Client Satisfaction Questionnaire. CEQ: *Credibility and Expectancy Questionnaire* AMPS: *Applied Mindfulness Process Scale*. CRS-PG: *Caregiver Reaction Scale – Personal Gain subscale HADS* A - D: *Hospital anxiety and depression scale.* UCLA_LS: *UCLA Loneliness Scale;* DERS: *difficulties in Emotion Regulation Scale. SCS: Self-Compassion Scale. MBPC: Memory and Behavior Problems Checklist. PAC: Positive Aspects of Caregiving. KAMU: Knowledge Assessment about music use.* CMAI*: Cohen–Mansfield Agitation Inventory.* AES*: Acceptability E-Scale.* DASS-21*: Depression Anxiety Stress Scales – Short Form. NIL&TS: Northern Ireland Life and Times Survey*. MSPSS*: Multidimensional Scale of Perceived Social Support.* EQ-5D-5L: EuroQol 5-Dimension 5-Level. SSCQ: Short Sense Of Competence Questionnaire. *Respite: ad-hoc self-report;* PROMIS: Promis Anxiety short-form questionnaire for adults; ZBISF-12*: Zarit burden short form 12;* MCSI: *Modified caregiver strain index*; CSAQ: *caregiver self- assessment questionnaire;* MCCWP-3: *3-item scale for measure caregiver communication with providers developed by Lorig et al., 1996.* PHQ-4: *Patient Health Questionary-4*; ADKS: *Alzheimer's Disease Knowledge Scale;* ZBI-22: *Zarit Burden Interview 22 items;* CDR: *Baseline clinical dementia rating*; NPI: *Neuropsychiatric Inventory*; CES-D: *Center for epidemiologic studies depression scale;* DCQI: *Dementia care quality indicators;* IFCN: *investigation and fulfillment of care needs;* ABS: *Abe's BPSD (behavioral and psychological symptoms of dementia) Score; J-ZBI-22: Japanese version of the Zarit Caregiver Burden Interview;* NPI-CD: *Neuropsychiatric inventory-caregiver distress;* SUS: *System usability scale;* BIQ: *Behavioral Intention Questionnaire-BCN acceptance ;* SWLS: *Satisfaction with life scale;* SSS: *Social support scale;* DKAT2: *Dementia knowledge assessment tool 2;* SF-12: *12-Item short form health survey;* PSS-10: *Perceived stress acale-10;*MARS-SQS*: MARS (mobile app rating scale) subjective quality section;* PHQ-9: *Patient health questionnaire-9;*SRS: *Self-Rated Health;* mMOS-SS: *Modified Medical Outcomes Study Social Support Survey;* WHOQOL-BREF: *World Health Organization Quality of Life-BREF; ;* eHEALS: *eHealth Literacy Scale;* BSFC: *Family Caregiver Burden Scale;* Brief-COPE: *Coping with problems experienced with caregivers;* WHO-5: *World Health Organization-Five Well-Being Index;* EMAs: *Daily ecological momentary assessments;* MARS: *Mobile Application Rating Scale;* QIDS: *Quick Inventory of Depressive Symptomatology;*

PANAS: *Positive and Negative Affect Schedule.* Ah-C-PwDRQ: *Ad hoc Caregiver–Person with Dementia Relationship Questionnaire;* PWBQ: *Psychological Well-Being Questionnaire (15 items) based on Ryff and Keyes*
