# Supplementary material for: mHealth To Promote Monitoring and Self‐Regulation Among Caregivers of People With Dementia: A Systematic Review
Source: Psych J. 2026 Apr 5;15(2):e70092. doi: 10.1002/pchj.70092 (PMC13052052; doi:10.1002/pchj.70092)
Supplement: Supplementary file 1 — Figure S1: Traffic Light Risk of Bias Using ROB‐2. Table S1: Studies' Exclusion Criteria and Corresponding Cohen's Kappa Inter‐rater Reliability. Table S2: Intervention Variables and Measurement Scales. Table S3: Guide to Determine the Level of Monitoring in an Intervention. Table S4: Features of the Selected mHealth Apps: Content, Monitoring and Outcomes. [file PCHJ-15-e70092-s001.zip › Table S3.pdf]

TABLE S3

Guide to Determine the Level of Monitoring in an Intervention

Dimension	Guiding Questions	Rating Options
**1. Presence of Monitoring**	Does the intervention include explicit monitoring activities (tracking, observing, recording progress)?	**None / Low / Medium / High**
**2. Type of Monitoring**	What kind of monitoring is implemented? – *External*: dashboards, automated tracking, alerts. – *Internal*: self-reflection, logging, mindfulness-based awareness.	**External / Internal / Both / None**
**3. Frequency & Intensity**	How often does monitoring occur? Daily? Weekly? Only at milestones? Is it integrated into routine use?	**High (daily/continuous)** / **Medium (weekly)** / **Low (occasional)**
**4. Feedback Generated**	Does the system or coach provide feedback based on monitored data? Is feedback automated, human, or reflective?	**None / Basic / Specific / Personalized**
**5. Use of Monitoring for Adjustment**	Are monitored data used to adapt goals, strategies, or recommendations?	**None / Minimal / Partial / Systematic**
**6. Responsibility for Monitoring**	Who performs monitoring? The system, user, caregiver, or professional? Is it passive (automatic) or active (user-driven)?	**System / User / Caregiver / Professional / Mixed**
**7. Overall Monitoring Level**	Integrated synthesis of previous criteria to classify the intervention's monitoring strength.	**Absent / Low / Medium / High**
