# Supplementary material for: mHealth To Promote Monitoring and Self‐Regulation Among Caregivers of People With Dementia: A Systematic Review
Source: Psych J. 2026 Apr 5;15(2):e70092. doi: 10.1002/pchj.70092 (PMC13052052; doi:10.1002/pchj.70092)
Supplement: Supplementary file 1 — Figure S1: Traffic Light Risk of Bias Using ROB‐2. Table S1: Studies' Exclusion Criteria and Corresponding Cohen's Kappa Inter‐rater Reliability. Table S2: Intervention Variables and Measurement Scales. Table S3: Guide to Determine the Level of Monitoring in an Intervention. Table S4: Features of the Selected mHealth Apps: Content, Monitoring and Outcomes. [file PCHJ-15-e70092-s001.zip › Table S4.pdf]

Table S4

Features of the Selected mHealth Apps: Content, Monitoring and Outcomes

Authors and Year	App Contents	Monitoring-related content	Caregiver Outcomes
Blackberry et al. (2023)	**Verily Connect**: 12 guides for caregivers (e.g. community life, keeping yourself well, taking a break), sources and directory of locasl services displayed in Google maps; videocoference	Permanent static content; Interaction between care managers and carers. External usage monitoring	Significant improvement in Social support (p = 0.003, Cohen's d = 0.61)<br>No differences in Burden (p = 0.09, Cohen's d = −0.33).
Castillo et al. (2023)	**Dementia Talk:** Dementia Information, Behavior Tracking, and Stress Management<br>**CLEAR Dementia Care:** Dementia Information, Care Suggestions, and Recording of Observed Behaviors	Permanent static content; synchronous contact; Video tutorial and optional initial Zoom (download and use App)	No significant differences in EG and GC in ZBI-22. Significant effects pre/post-test: PSS-10; ZBI-22 (p <0.01); and DKTA2 (p <0.01)
Coleman et al. (2025)	TLC: Self-guided digital tool ("Time for Living and Care") that employs automated coaching to support caregivers in planning and evaluating respite time	Respite planning goal setting, barrier–strategy identification, and automated coaching. Dashboard that tracks respite hours, progress, and weekly goal-review prompts, alongside burden and health monitoring	Significant improvement in positive aspects of care; significant moderation of caregiving time (p < .001). Greater effect among those providing over 80% of care
Collins-Pisano et al. (2024)	**CARES:** Caregiver remote re-education and support, contents peer support, coping skills, psychoeducation, medical management, social skills, and self-advocacy and self-management	Permanent static content; peers support; app messaging; videos; Specific intervention on recognizing negative thoughts	Non-significative differences on Burden Straind and Strees. Effect size from 0.44 (stress), 0.44(Burden), and 0.13 (Strain)

Table S4

Features of the Selected mHealth Apps: Content, Monitoring and Outcomes

Authors and Year	App Contents	Monitoring-related content	Caregiver Outcomes
Gallegos et al. (2025)	Headspace: modules on mindfulness, reframing loneliness, and self-compassion; guided breathing app	Internal monitoring of thoughts and emotions in mindfulness	No effects in the experimental group
Goto et al. (2024)	Information on the care: community-based integrated care systems and miscellaneous topics related to caregiving) and exchanging information on how to care as social network	Permanent static content; virtual peers contact; face to face preliminary assessment; monthly assessment of BPSD and Burden along four month	Burden significantly decreased following the use of the web application (p = 0.013)
Goodridge et al. (2021)	**Ethica:** 14 podcasts, 12 meditations, and 4 body practices (MBSC) + guidelines for communication, emotional management, and stress and fatigue management	Permanent static content; app messaging (EMA: momentary ecological assessment "How are you feeling today?") Instructions on use App	Significant differences pre/post-test in WHO-5 (p=0.04) and emotion-based coping Brief-COPE (p=0.01). No significant differences in Burden
Hong et al. (2023)	**WECARE:** Dementia Knowledge, Skills, Effective Communication, Problem Solving, Stress Reduction and Depression Prevention, Self-Care and Health and Social Support and Local Resources	Permanent static content; synchronous contact; Multimedia articles sent to WeChat (RRSS) weekly + group; private chats and peer-to-peer support video calls + Initial Zoom (consent and doubts App) + 3 moderated group sessions	Significant pre/post-test differences in: CES-D (p<0.001); ZBI-22 (p = 0.03). SWLS (p=0.02). No significant differences in EFS (p= 0.51)
Hong et al. (2024)	**Virtual passport**: dementia management quality measurement set and care needs (e.g. education, functional status assessment, pain assessment)	Permanent static content; Interaction between care managers and carers; emphasis on needs identification	Burden significantly decreased at six (p = >.09) and 12 month (p = >.09) comparing with control group. Detection of needs enhanced after six .37 vs 0, p<0.001) and 12 month (1.32 vs 0, p<0.001). Significant

Table S4

Features of the Selected mHealth Apps: Content, Monitoring and Outcomes

Authors and Year	App Contents	Monitoring-related content	Caregiver Outcomes
			improvement in irritability/liability (0.58 vs 0.22, p=0.044) and agitation/aggression after the intervention -0.78 vs 0.00, p=0.042).
Iacob et al. (2024)	TLC: Self-guided digital tool ("Time for Living and Care") that employs automated coaching to support caregivers in planning and evaluating respite time	Respite planning goal setting, barrier–strategy identification, and automated coaching. Dashboard that tracks respite hours, progress, and weekly goal-review prompts, alongside burden and health monitoring	Significant increases in respite time, scheduling, and satisfaction ($p < .001$). Anxiety increased with access to the calendar, decreased with weekly coaching $p < .001$, and rose again when the intervention was removed ($p = .008$)
Kagwa et al. (2025)	STAV: Chat with professionals; digital diary; mindfulness sessions; service contacts; information links.	Personal diary, professional monitoring	No significant impact on Burden or depression
Leung et al. (2022)	**e-painting:** Electronic painting, image sharing function, bulletin board and self-assessment	Permanent static content; peers contact; app messaging; face to face contact	No significant differences in PHQ-9, SSR, mMOS-SS. Significant increase in ZBI p=0.004. Frequency of use correlated with the exchange EG of paints with friends or family ($r=0.72$, $p <0.001$). Qualitative: satisfaction, enjoyment in the use of the app and channel to express their emotions and connect with other caregivers.
Neal et al.(2024)	FindMyApps: Search and recommendation tool for apps rated as easy to use by people with dementia	-	No significant impact on quality of life. Significantly greater sense of competence (+0,75; IC 95% 0,14 – 1.3)

Table S4

Features of the Selected mHealth Apps: Content, Monitoring and Outcomes

Authors and Year	App Contents	Monitoring-related content	Caregiver Outcomes
Nguyen et al. (2025)	Psychoeducational program delivered via the Zalo messaging app; contact with professionals	Chat questions about doubts and offers informal self- and qualitative evaluation of progress and difficulties.	Significant ($p < .001$) improvements in depression, anxiety, stress, knowledge, burden, and social support, sustained at three months
Park et al. (2020)	**CMAP:** Understanding Dementia, Pharmacological and Non-Pharmacological Treatments, Environmental Management, Communication Skills, SPCD Response Methods, and Bulletin Board	Permanent static content; professional contact; weekly calls; face to face contact	Significant differences between EG and CG in ZBI (χ 2 = 18.17, p<0.001) and E. Piper (F = 11.24, p = 0.003). No differences in Cortisol, E.Lee and NPI
Plys et al. (2025)	HMP-C (Healthy Minds Program for Caregivers). Mindfulness intervention based on awareness, connection, insight, and purpose	Reminders, motivational messages, adherence-triggered calls, listening history, external monitoring, and mindfulness self-observation	Significant improvement in the mindfulness process and in the appraisal of positive aspects of caregiving ($p < .001$), with no impact on stress, anxiety, or depression
Rodriguez et al. (2023)	**Brain CareNotes:** Weekly NPI-based assessments to automatically receive a set of CareNotes with "short" tips	Permanent static content; professional contact; app messaging; app training; phone or email support and help manuals. Self-Assessment and Reports.	Statistical differences in NPI measures between EG y CG at baseline and 3 months, but disappeared by 6 months.
Romero-Mas et al. (2021)	**Estic amb tu** [I'm with you]: A space for the exchange of knowledge between caregivers, member directory, community management	Permanent static content; app messaging; peer support; face to face contact	Significant differences between EG1 and EG2 pre/post-test in WHOQOL-BREF (p=0.002).

Table S4

Features of the Selected mHealth Apps: Content, Monitoring and Outcomes

Authors and Year	App Contents	Monitoring-related content	Caregiver Outcomes
Ruggieri et al. (2024)	**CareHeroes**: Provider communication, health literacy, educational information and chatbot, clinical information about the care recipient, clinical evaluation form for the patient and care, feedback.	Permanent static content; 3 Professional contact; interaction AI chatbot; feedback; self assessments (burden, health)	Reduces depression at 3 month (p = 0.3). 169 actions used, 26% of them with chatbot.
Sikder et al. (2019)	**MIT App:** MIT Audio Guide (Stretching and Breathing, Eye in the Center, Russian Doll, Problem Solver, Globe of Life) and 4 case studies (1/week) use of the techniques	Permanent static content; Daily reminders; optional initial call	Significant pre/post differences in depression (p=0.008); increased positive moods (p<0.05). Qualitative: "calm," "reduced stress," and an "anchor" for your mood
Smith et al. (2025)	Real-time location set geofenced safe zones, receive alerts, and use quick two-way calling for immediate communication	Real-time location set georeferenced safe zones	Higher caregiver burden was linked to better app perception.
Thompson et al. (2025)	MATCH: app for using music in daily activities and during agitation	Weekly calls to monitor app outcomes. Diary recording	Significant reduction (p < .001) in: distress, in knowledge about strategic music use.
Watcharasarnsap et al. (2020)	Natural Language Processing; for reminiscence therapy	-	Significant differences between EG and CG in Relationship (P=0.458, α = 0.0115) and Psychological well-Being (P=0498, α = 0.004)
